# Exposure to prenatal secondhand smoke and early neurodevelopment: Mothers and Children’s Environmental Health (MOCEH) study

**DOI:** 10.1186/s12940-019-0463-9

**Published:** 2019-03-20

**Authors:** Myeongjee Lee, Mina Ha, Yun-Chul Hong, Hyesook Park, Yangho Kim, Eui-Jung Kim, Yeni Kim, Eunhee Ha

**Affiliations:** 1 Department of Occupational and Environmental Medicine, School of Medicine, Ewha Womans University, Seoul, Republic of Korea; 2 Department of Preventive Medicine, College of Medicine, Dankook University, Cheonan, Republic of Korea; 3 Institute of Environmental Medicine, Medical Research Center, Seoul National University, Seoul, Republic of Korea; 4 Department of Preventive Medicine, School of Medicine, Ewha Womans University, Seoul, Republic of Korea; 5 Department of Occupational and Environmental Medicine, Ulsan University Hospital, University of Ulsan College of Medicine, Ulsan, Republic of Korea; 6 Department of Psychiatry, School of Medicine, Ewha Womans University, Seoul, Republic of Korea; 7 Department of Child and Adolescent Psychiatry, National Center for Mental Health, Seoul, Republic of Korea; 8 Ewha Medical Research Institute, School of Medicine, Ewha Womans University, Seoul, Republic of Korea

**Keywords:** Secondhand smoke, Urine cotinine, Infant neurodevelopment, Genetic polymorphism, Breastfeeding, 24 months

## Abstract

**Background:**

The association between exposure to secondhand smoke (SHS) during pregnancy and a child’s neurodevelopment has not been established yet. We explored the association between prenatal exposure to SHS and neurodevelopment at 24 months of age considering genetic polymorphism and breastfeeding in 720 mothers and their offspring enrolled in the Korean multicenter birth cohort study (Mothers and Children Environmental Health, MOCEH).

**Methods:**

We quantified urine cotinine concentrations in mothers once from 12th to 20th gestational weeks and excluded those whose urine cotinine levels exceeded 42.7 ng/ml to represent SHS exposure in early pregnancy. Mental developmental index (MDI) and psychomotor developmental index (PDI) values were measured using the Korean version of the Bayley Scales of Infant Development II (K-BSID-II) at 24 months of age. A general linear model was used to assess the relationship between maternal urinary cotinine level and neurodevelopment.

**Results:**

MDI scores were inversely associated with cotinine [β = − 2.73; 95% confidence interval (CI): − 5.32 to − 0.15] in children whose mothers had early pregnancy urinary cotinine levels >1.90 ng/ml. No association was evident in children whose mothers had cotinine levels ≤1.90 ng/ml. This negative association was more pronounced in children whose mothers had both Glutathione S-transferases mu 1 (GSTM1) and theta 1 (GSTT1) null type [β = − 5.78; 95% CI: -10.69 to − 0.87], but not in children whose mothers had any present type of GSTM1/GSTT1 [β = − 1.64; 95% CI: -4.79 to 1.52]. The association was no longer significant when children received breast milk exclusively for up to 6 months [β = − 0.24; 95% CI: -4.69 to 4.20] compared to others [β = − 3.75; 95% CI: -7.51 to 0.00]. No significant association was found for PDI.

**Conclusions:**

Maternal exposure to SHS during pregnancy may result in delayed MDI in early childhood. This effect might be modified by genetic polymorphism and breastfeeding behavior.

**Electronic supplementary material:**

The online version of this article (10.1186/s12940-019-0463-9) contains supplementary material, which is available to authorized users.

## Background

Exposure to active smoking and secondhand smoke (SHS) causes health concern. About 40% of children, 35% of women, and 33% of men are exposed to SHS in their daily lives [1]. World Health Organization (WHO) has recently reported that environmental risks including SHS take lives of 1.7 million children under 5 years of age every year [2]. Harmful exposure to these environmental risks could begin in the mother’s womb and affect fetal development. Therefore, more attention should be paid to pregnant women and infants who are susceptible to SHS exposure.

It is well known that SHS exposure brings about almost the same adverse health outcomes as active smoking [3]. Smoking during pregnancy is a well-known risk factor for adverse birth outcomes such as spontaneous abortion [4], low birth weight, and preterm birth [5] that, in turn might affect children’s development. Tobacco smoke contains over 7000 chemicals including nicotine, polycyclic aromatic hydrocarbons (PAHs), aromatic amines, and carbon monoxide. Placental passage of these environmental toxicants might affect prenatal nervous system development. Although effects of prenatal exposure to SHS on early neurodevelopment vary among studies, they remain significant issues. Lower development scores in cognition, language, and fine motor scales [6, 7], gross motor scores [8], and MDI scores [9] have been reported in children with prenatal SHS exposure. Therefore, SHS exposure should be considered a modifiable risk factor for delayed neurodevelopment and cognitive impairment in children. As cotinine is a predominant metabolite of nicotine, it is considered a biomarker of exposure to SHS [10]. Cotinine assays provide an objective quantitative measure that is more reliable than smoking history or counting the number of cigarettes smoked per day. Measure of cotinine in hair, blood, and urine permits the assessment of SHS exposure or active smoking.

Metabolic gene polymorphisms might modify the effect of toxins on the outcome of pregnancy and development afterwards. Glutathione S-transferases mu 1 (GSTM1) and theta 1 (GSTT1) are major detoxification phase II enzymes that provide critical defense against xenobiotics. Numerous smoke-derived chemicals such as PAHs and aromatic amines can be detoxified by GSTM1. Metabolites of 1,3-butadiene and ethylene oxide present in tobacco smoke are detoxified by GSTT1 [11]. Homozygous deletion polymorphisms of GSTM1 and GSTT1 (GSTM1-null and GSTT1-null) can result in loss of function and increase the risk of faulty fetal development [12]. GSTM1/GSTT1 polymorphisms can significantly modify birth outcomes following maternal exposure to tobacco smoke [13–16], heavy metals [17], perfluorinated compounds [18], and particulate matter [19]. These polymorphisms also influence early neurodevelopment of infants born following maternal exposure to environmental tobacco smoke [6].

Breastfeeding is thought to be associated with better neurodevelopment. It is well known that breastfeeding provides nutritional and immunological benefits to infants and promotes cognitive development between the mother and the infant [20–22]. Improved cognitive development of infants who are breastfed longer has been described elsewhere [23]. Benefits of breastfeeding even extend to mothers who are current smokers [24].

Although the Korean government has established several public policies since 1995, 39.7% of non-smokers are still exposed to SHS. This percentage is higher than that in other countries. In addition, 18.5% of pregnant women responded affirmatively to SHS exposure in Korea [25]. Although concern about the effect of SHS on health has been increasing, very few researches have been conducted using Korean population.

Therefore, the aim of this study was to investigate the association between prenatal SHS exposure and neurodevelopment of infants 24 months after birth using Korean birth cohort study. We further identified whether the association could be modified by maternal genetic polymorphisms and breastfeeding behavior.

## Methods

### Study population

**Fig. 1:**
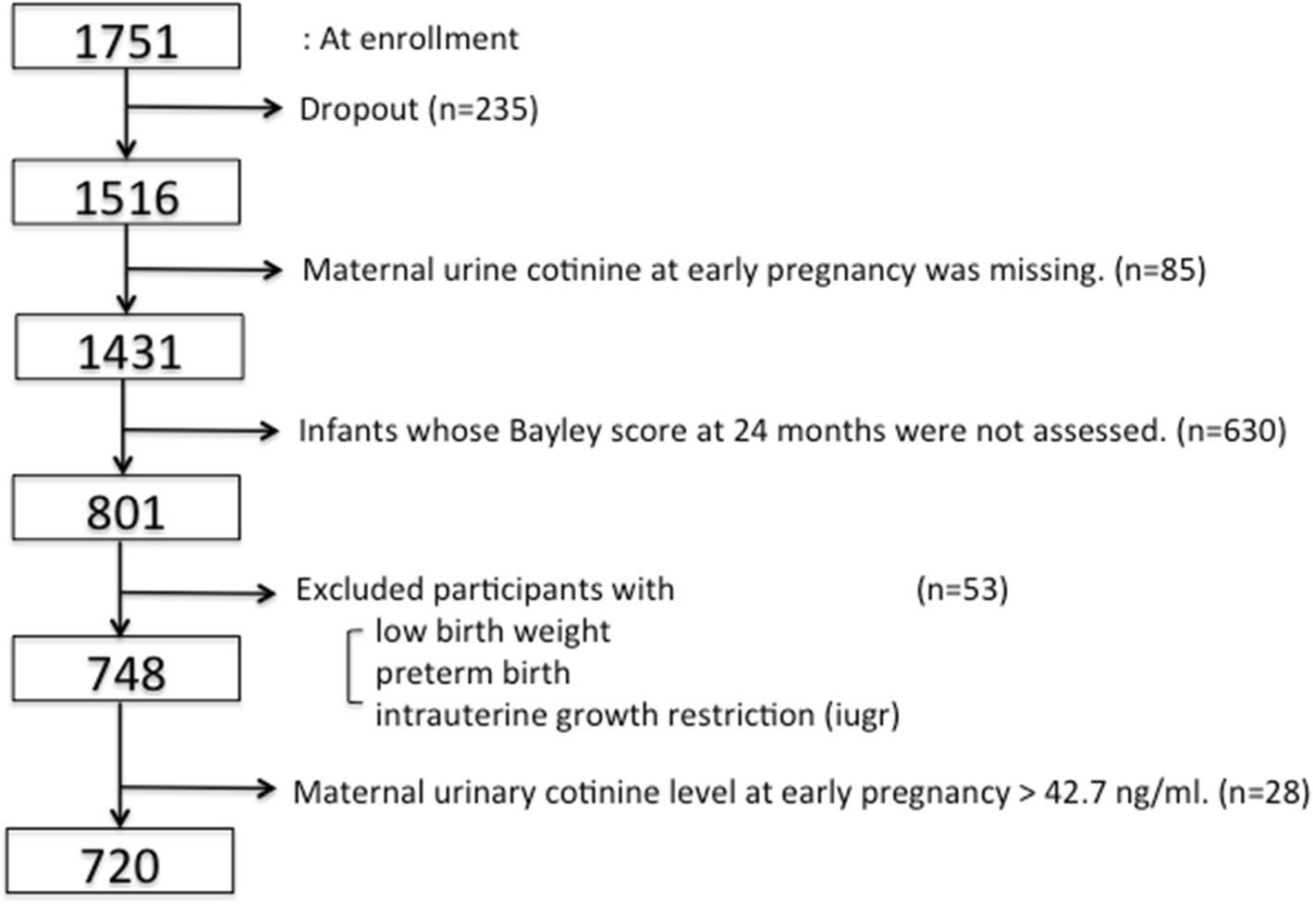
Flowchart showing the selection of the study population at 24 months (*n* = 720)

Data were from the Mothers and Children’s Environmental Health (MOCEH) study, a multicenter prospective cohort study initiated since 2006 in Korea. This study was designed to collect information related to environmental exposures during pregnancy and childhood to examine how exposure to environmental pollutants might affect growth, development, and disease in South Korea. Detailed information of the MOCEH study has been described previously [26]. Pregnant women in their first trimester were recruited from three university hospitals located in Seoul (metropolitan area), Cheonan (urban area, midwest), and Ulsan (metropolitan and industrial area, southeast) between 2006 and 2010. Participants were > 18 years of age. A total of 1751 pregnant women were enrolled in the beginning and 1516 mother-child pairs were followed up after birth (Fig. 1). Our study subjects were restricted to 801 children whose maternal urinary cotinine levels were determined between 12th and 20th gestational weeks. They also completed a neurodevelopmental follow-up assessment using Bayley tests at 24 months of age. There were no significant differences between children with Bayley scores (*n* = 801) and those without Bayley scores (*n* = 630) at 24 months with respect to maternal age, mother’s education level, genetic polymorphisms, sex of children, or breastfeeding behavior except for residential area and the primary caregiver up to 24 months after birth (Additional file 1: Table S1). Children (*n* = 53) were excluded if they were born with low birth weight (birth weight < 2500 g), preterm (gestational age < 37 weeks), or diagnosed with intrauterine growth restriction at birth. Written informed consent was obtained at the initial visit from all enrolled mothers on behalf of themselves and their children. Study protocols were approved by Institutional Review Boards of Ewha Womans University (Seoul), Dankook University Hospital (Cheonan), and Ulsan University Hospital (Ulsan).

### Measurement of urine cotinine levels

Cotinine is considered a good biomarker of tobacco-smoke exposure. Urine can be obtained easily and non-invasively from pregnant women. Thus SHS exposure during pregnancy was quantified using urinary cotinine level in this study. Maternal urine was sampled once from 12th to 20th gestational weeks. Cotinine Direct ELISA Kits (Calibiotech, Spring Valley, CA, USA) were used for determinations. Urine was diluted 1:100 and 10 μl of the diluted sample was applied in duplicates to 96-well microtiter plates. Diluted urine sample was incubated with 100 μl of enzyme conjugate at room temperature for 60 min. Wells were washed six times with 300 μl distilled water prior to addition of 100 μl of substrate reagent to each well. Plates were then incubated at room temperature for 30 min. Absorbance was measured at 450 nm on an ELISA reader within 15 min after adding 100 μl of Stop Solution. Limit of detection (LOD) for urinary cotinine was 1.0 ng/ml. Values below this limit were converted to non-negative values by half of the detection limit (LOD/2) in the analysis. Urinary creatinine levels were adjusted in our analyses.

### Assessment of infants’ MDI and PDI at 24 months

Neurodevelopment of infant at 24 months of age was measured using the Korean version of Bayley Scale of Infant Development II (K-BSID-II) [27], a standardized tool for assessing infant neurodevelopment [28]. This produces developmental indices and composite scores to compare developmental performance of a child with norms taken from typically developing Korean children of the same age. K-BSID-II also assesses habituation, problem solving, memory, classification, vocalization, and language skills. The resulting score represents mental development index (MDI). Psychomotor development index (PDI) is scored for the degree of body control, muscle coordination, postural control, and finer manipulatory skills. K-BSID-II has shown excellent test-retest stability and inter-rater agreement [27]. Test score was standardized, having a mean of 100 and a standard deviation of 15 [29]. To increase the inter-rater consistency, annual rater training sessions with video monitoring were held (inter-rater consistency: kappa value > 0.8) [30].

### Genotyping of GSTM1 and GSTT1

Genomic DNA was extracted from maternal whole blood using a QIAamp DNA blood kit (Qiagen, Valencia, CA, USA). Polymerase chain reaction (PCR) was used to genotype GSTM1 and GSTT1 polymorphisms. As a positive control, a 268-bp fragment of β-globin gene was amplified at the same time. PCR mixture (20 μl) for GSTM1 and GSTT1 genotyping contained 10 mM Tris-HCl (pH 9.0), 40 mM KCl, 1.5 mM MgCl_2_, 0.25 mM of each dNTP, 1 unit Taq polymerase (Bioneer, Seoul, Korea), 20 pmol of forward and reverse primers each, and 50–100 ng of the genomic DNA as a template. The following primer sets of GSTM1 and GSTT1 genes were used for PCR reaction: 5′-GAACTCCCTGAAAAGCTAAAGC-3′ (forward) and 5′-GTTGGGCTCAAATATACGGTGG-3′ (reverse) for GSTM1, and 5′-TCACCGGATCATGGCCAGCA-3′ (forward) and 5′-TTCCTTACTGGTCCTCACATCTC-3′ (reverse) for GSTT1. Amplifications were performed using an initial denaturation at 94 °C for 5 min; 35 cycles of denaturation at 94 °C for 1 min, annealing at 65 °C for 1 min, and extension at 72 °C for 1 min; and a final extension at 72 °C for 7 min. PCR amplification of the reaction mixture was carried with a PTC-200 thermal cycler (MJ Research, Watertown, MA, USA).

To evaluate PCR-amplified fragments, electrophoresis was performed using 3% 3:1 NuSieve/agarose gel (Cambrex Bio Science, Rockland, ME, USA). Genotyping of GSTM1 and GSTT1 genes was performed based on the presence of a 215-bp product and a 459-bp product, respectively. Null genotype was defined as a homozygous deletion of the gene. To confirm results of analyses, 10% of samples were randomly selected and genotyped again, with identical results.

### Covariates

Potential confounders were selected through literature review. Sociodemographic data including maternal age, maternal education level, and region were reported in the baseline questionnaire at the first visit. Each variable was considered as a categorical variable: ≤ 30 years old or > 30 years old for maternal age; ≤ high school or ≥ university for maternal education; and Seoul, Cheonan, and Ulsan for region. Information of infant sex as a categorical variable and gestational age as a continuous variable was obtained at delivery. At 6-month follow-up, mothers were asked about how they were feeding their babies. We categorized children into two groups: breastfeeding only for up to 6 months as exclusive breastfeeding or others. We also defined the primary caregiver during the first 24 months after birth based on the questionnaire at each follow-up: mother during the whole period or others.

### Statistical analyses

First, we conducted receiver operating characteristic (ROC) curve analysis [31] to obtain the optimal cut-off point of urinary cotinine levels for distinguishing active smokers from non-active smokers during pregnancy. Based on our database, active smoker was defined as a mother who responded positively to a question, ‘Do you smoke now?’ in early or mid-pregnancy. Everyone else was considered a non-active smoker. ROC analysis is a graphical and quantitative technique that can determine the optimal cut-off point for a classified decision for a given continuous criterion variable. Here, the criterion variable is urinary cotinine. The classified condition is self-reported smoking status during pregnancy. Sensitivity is the percentage of smokers exceeding the cut-off point and specificity represents the percentage of nonsmokers below the cut-off point.

Characteristics of the study population are presented as mean ± standard deviation (SD) for continuous variables or numbers and percentages for categorical variables. Difference was compared using Chi-squared test or t-test. Maternal urinary cotinine level was natural log (ln)-transformed in all analyses because of its skewed distribution. Summary statistics for urinary cotinine level (including arithmetic and geometric means, and percentiles) were calculated.

Generalized additive model (GAM) [32] was used to examine the non-linear association between maternal urinary cotinine level at early pregnancy and offspring’s K-BSID-II test score at 24 months. Based on results from GAM analysis, children were divided into two groups according to their mothers’ urinary cotinine levels: above or below the median (1.90 ng/ml). For each group, associations between maternal urinary cotinine levels and offspring’s K-BSID-II test scores were separately assessed using a multiple linear regression model after adjusting for ln-transformed urinary creatinine level, sex of a child, maternal age, maternal education, gestational age, region, breastfeeding up to 6 months, and primary caregiver during the first 24 months after birth. Data were further analyzed after data stratification by maternal genetic polymorphisms or breastfeeding to investigate their modification effect on the association. Maternal GSTM1/GSTT1 genotype was classified as any present or both null. We then tested the significance of interaction term between maternal urinary cotinine level at early pregnancy and each stratum.

A supplementary analysis was conducted to examine the robustness of results. We repeated all analyses without adjusting for urinary creatinine because using urine cotinine values not corrected for urine creatinine values showed high correlation with smoking behavior [33]. All analyses were considered to be statistically significant if *p*-values were less than 0.05. All data preparation and analyses were performed using SAS statistical package, version 9.4 (SAS Institute Inc., Cary NC, USA).

## Results

**Fig. 2:**
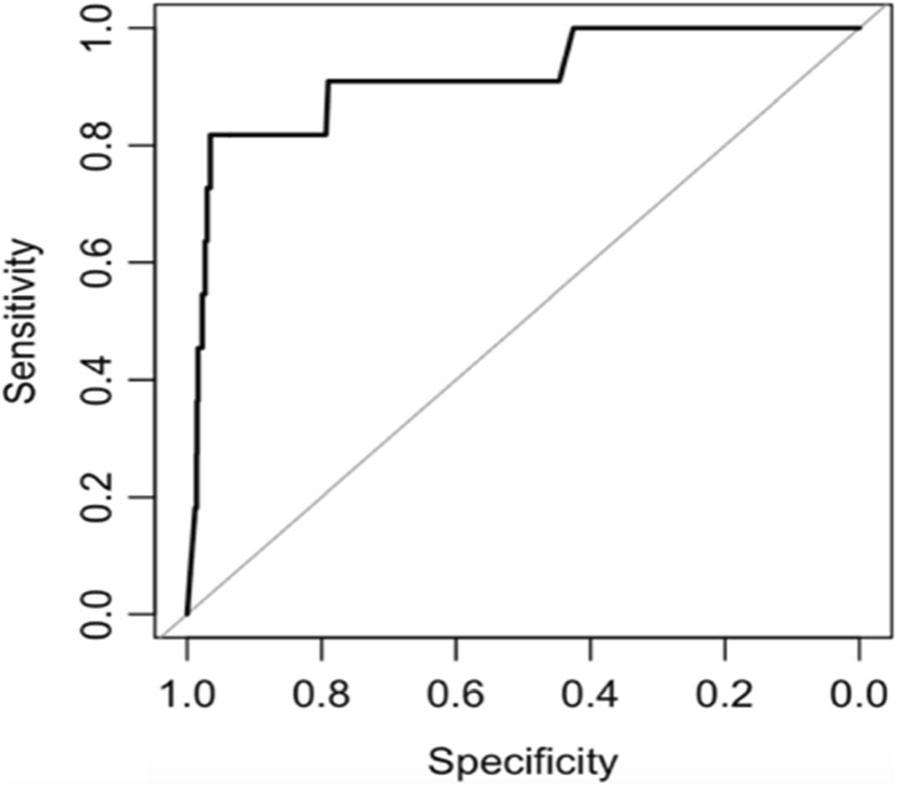
ROC curve analysis of urinary cotinine levels for differentiating current smokers from nonsmokers. The optimal urine cotinine cut-off level to distinguish active smokers from non-active smokers was 42.7 ng/ml, with sensitivity of 81.8% and specificity of 96.5%. The area under the curve (AUC) was 0.91

From ROC curve analysis, the optimal cut-off level for discriminating active smokers from non-smokers during pregnancy was 42.7 ng/ml, with a sensitivity of 81.8%, a specificity of 96.5%, and an area under the curve of 0.91 (Fig. 2). We then further excluded 28 children whose mothers were considered as active smokers because their urine cotinine levels exceeded 42.7 ng/ml. Finally, 720 mother-child pairs were included in the our analyses.

Demographic characteristics of study participants are presented in Table 1. Sixty percent of the women were ≤ 30 years old and 51.7% had at least a university education. MDI scores at 24 months were significantly higher in children with younger mothers. MDI and PDI scores at 24 months varied significantly according to mother’s education level and region. Girls displayed significantly higher scores of MDI and PDI at 24 months than boys. The median of maternal urinary cotinine level assessed at early pregnancy was 1.90 ng/ml.

**Table 1:** Characteristics of study population

	Number (%) or Mean ± SD		at 24 months			
		*P*	MDI		PDI	
			Mean ± SD	*P*	Mean ± SD	*P*
Total	720		97.01 ± 14.52		96.78 ± 13.50	
Maternal characteristics						
Maternal age, years						
≤30	429 (59.6)	<0.001	98.28 ± 14.45	0.004	97.00 ± 13.57	0.59
>30	291 (40.4)		95.13 ± 14.44		96.45 ± 13.42	
Maternal education						
≤High school	284 (39.4)	<0.001	95.23 ± 14.60	0.03	94.64 ± 13.74	0.002
≥University	372 (51.7)		98.16 ± 14.40		98.04 ± 13.31	
missing	64 (8.9)		98.25 ± 14.27		98.97 ± 12.42	
Region						
Seoul	177 (24.6)	<0.001	95.51 ± 14.43	0.02	97.97 ± 13.03	<0.001
Cheonan	340 (47.2)		96.34 ± 15.20		93.53 ± 12.84	
Ulsan	203 (28.2)		99.44 ± 13.14		101.19 ± 13.63	
GSTM1						
Present (=positive)	315 (44.4)	0.003	96.19 ± 14.10	0.22	95.88 ± 12.66	0.12
Null (=negative)	394 (55.6)		97.53 ± 14.92		97.47 ± 14.20	
GSTT1						
Present (=positive)	345 (48.7)	0.48	96.32 ± 14.50	0.28	96.30 ± 13.25	0.38
Null (=negative)	364 (51.3)		97.51 ± 14.62		97.20 ± 13.84	
Urinary cotinine						
≤median^a^	368 (50.6)	0.74	97.05 ± 14.81	0.80	96.86 ± 14.07	0.80
>median^a^	359 (49.4)		96.77 ± 14.43		96.60 ± 12.97	
Infant characteristics						
Infant sex						
Male	381 (52.9)	0.12	92.56 ± 14.31	<0.001	94.81 ± 13.45	<0.001
Female	339 (47.1)		102.01 ± 13.07		99.00 ± 13.23	
Breastfeeding only up to 6 months						
Yes	257 (35.7)	<0.001	98.81 ± 14.40	0.04	97.33 ± 14.30	0.11
No	383 (59.2)		95.87 ± 14.55		95.93 ± 13.04	
missing	80 (11.1)		96.68 ± 14.37		99.11 ± 12.81	
Primary caregiver during the first 24 months after birth						
Mother	368 (51.1)	0.55	97.90 ± 14.42	0.09	96.49 ± 13.99	0.56
Others	352 (48.9)		96.08 ± 14.58		97.09 ± 12.98	
Gestational Age	39.06 ± 1.08					

*SD* standard deviation, *MDI* Mental Development Index, *PDI* Psychomotor Development Index, *GSTM*1 Glutathione S-transferases mu1, *GSTT*1 Glutathione S-transferases theta 1

^a^Median of urinary cotinine level is 1.90 ng/ml

**Fig. 3:**
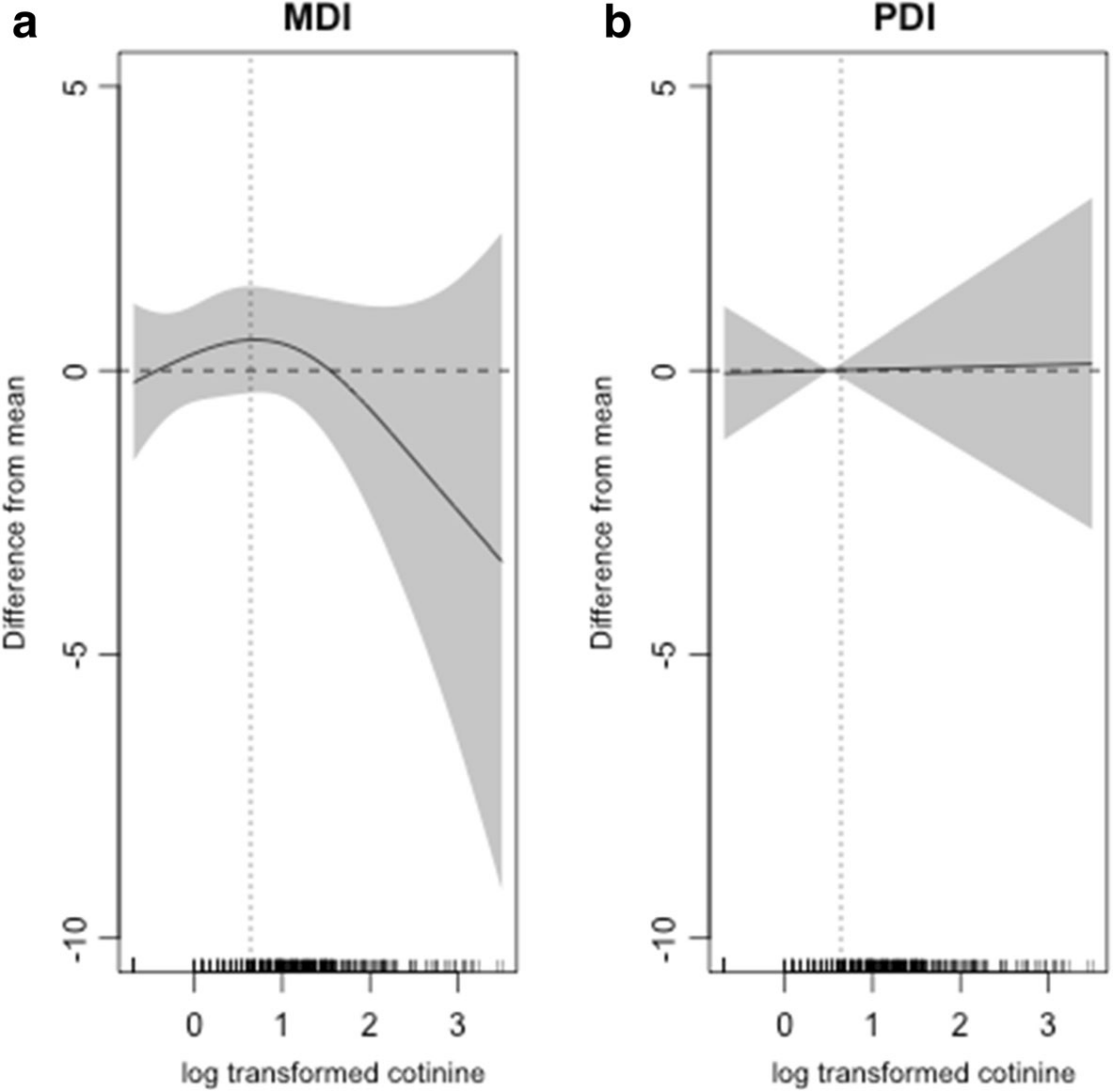
Unadjusted associations of ln-transformed maternal urinary cotinine levels and Bayley scores in infants at 24 months of age. **a** Mental Development Index (MDI) and (**b**) Psychomotor Development index (PDI). The Generalized Additive Model was used**.** Dashed vertical straight line at 0.64 in each figure represents the median level of urinary cotinine, exp. (0.64) = 1.90

Figure 3 presents a nonlinear relationship between ln-transformed maternal urinary cotinine level and residualized unadjusted MDI and PDI scores. Concerning MDI, no association was evident for lower level of urinary cotinine, although a negative association was observed for higher level of urinary cotinine (Fig. 3 (a)). Therefore, we divided our data into two cohorts and analyzed separately. One cohort included children whose maternal urinary cotinine levels at early pregnancy were at or below the median and the other cohort included children whose maternal urinary cotinine levels at early pregnancy were above the median. However, this pattern was not observed for PDI (Fig. 3 (b)).

MDI scores of infants at 24 months of age and maternal urinary cotinine levels during early pregnancy were negatively associated if maternal urinary cotinine level exceeded the median (β = − 2.73, 95% CI: -5.32 to − 0.15 from adjusted analysis) (Table 2). However, such negative association between infant’s PDI score at 24 months and maternal urinary cotinine level disappeared. No association was found when maternal urinary cotinine level in early pregnancy was lower than the median.

**Table 2:** Association between maternal urine cotinine in early pregnancy and children’s neurodevelopment at 24 months

Cotinine	Bayley	n	Mean ± SD	Unadjusted^a^			Adjusted^b^		
				β	95% C.I.	*P*	β	95% C.I.	*P*
≤ 1.90	MDI	368	97.05 ± 14.81	1.41	(−1.57, 4.38)	0.35	1.31	(−1.52, 4.14)	0.36
	PDI	368	96.86 ± 14.07	1.63	(−1.20, 4.46)	0.26	1.88	(−0.90, 4.66)	0.19
> 1.90	MDI	352	96.97 ± 14.24	−1.79	(−4.29, −0.71)	0.16	−2.73	(−5.32, − 0.15)	0.04
	PDI	352	96.70 ± 12.89	−0.60	(−2.87, 1.67)	0.61	− 0.35	(−2.69, 2.00)	0.77

^a^General Linear Model adjusted for creatinine

^b^General Linear Model adjusted for maternal age, maternal education, gestational age, infant sex, region, breastfeeding behavior, primary caregiver, and creatinine

*MDI* Mental Development Index, *PDI* Psychomotor Development Index

The effect of maternal urinary cotinine level at early pregnancy on infant’s MDI score at 24 months of age differed depending on genetic polymorphism and breastfeeding behavior (Additional file 1: Figure S1). The observed significant negative association from Table 2 became much stronger for infants whose mothers had GSTM1/GSTT1 double deletion (β = − 5.78, 95% CI: -10.69 to − 0.87) (Table 3). The significance of this negative association disappeared in infants whose mothers had high cotinine levels when infants received breastmilk exclusively up to 6 months of age (β = − 0.24, 95% CI: -4.69 to 4.20). However, we found that none of the interaction terms between urinary cotinine level and each stratum was statistically significant.

**Table 3:** Association between maternal urine cotinine in early pregnancy and children’s neurodevelopment at 24 months stratified by genetic polymorphism and breastfeeding behavior

	Cotinine	≤ 1.90^a^					> 1.90^a^				
		n	β^b^	95% C.I.	*P*	*P*_*interaction*_^d^	n	β^b^	95% C.I.	*P*	*P*_*interaction*_^d^
MDI	GSTM1/GSTT1										
	Any present	241	2.51	(−1.14, 6.16)	0.18	0.25	251	− 1.64	(−4.79, 1.52)	0.31	0.16
	Both null	119	−0.40	(−5.53, 4.74)	0.88		98	−5.78	(−10.69, − 0.87)	0.02	
	Breastfeeding only up to 6 months^3^										
	Yes	135	0.35	(−4.38, 5.09)	0.88	0.38	122	−0.24	(−4.69, 4.20)	0.91	0.22
	No	195	2.31	(−1.94, 6.56)	0.29		188	−3.75	(−7.51, 0.00)	0.05	
PDI	GSTM1/GSTT1										
	Any present	241	2.45	(−1.07, 5.98)	0.17	0.83	251	−0.42	(−3.35, 2.51)	0.78	0.87
	Both null	119	2.26	(−2.95, 7.46)	0.39		98	−1.25	(−5.64, 3.14)	0.57	
	Breastfeeding only up to 6 months^c^										
	Yes	135	−0.70	(−5.86, 4.46)	0.79	0.16	122	0.86	(−3.55, 5.27)	0.70	0.23
	No	195	3.29	(−0.51, 7.09)	0.09		188	−0.70	(−3.96, 2.56)	0.67	

^a^median of urinary cotinine level = 1.90 (ng/ml)

^b^General Linear Model adjusted for maternal age, maternal education, gestational age, infant sex, region, breastfeeding behavior, primary caregiver, and creatinine

^c^General Linear Model adjusted for maternal age, maternal education, gestational age, infant sex, region, primary caregiver, and creatinine

^d^*P*-value for interaction

*MDI* Mental Development Index, *PDI* Psychomotor Development Index, *GSTM*1 Glutathione S-transferases mu1, *GSTT*1: Glutathione S-transferases theta 1

These results did not change substantially when we conducted analyses without adjusting for urinary creatinine level (Additional file 1: Tables S2 and S3).

## Discussion

We explored the association between maternal SHS exposure during early pregnancy and infant neurodevelopment at 24 months of age. We also examined the effect of genetic polymorphism and exclusive breastfeeding on such association. A significant association was found in children whose mothers had higher (greater than median) levels of cotinine. Cognitive development of 24 months old infants decreased significantly with increasing maternal cotinine level. For mothers with both GSTM1 and GSTT1 null types, the negative association became stronger. An association between PDI of 24 months old infants and maternal SHS exposure early in the pregnancy was not evident.

Previous studies have investigated the impact of prenatal maternal SHS exposure on infants’ neurodevelopment. Secondhand smoke exposure during pregnancy could be measured by parental self-reports or biomarkers such as cotinine in cord blood and cotinine in maternal urine during pregnancy. Based on self-reported prenatal exposure to SHS, previous studies have shown a negative impact of such exposure on cognitive development of infants aged 6–36 months [9, 34, 35]. Such negative association has also been observed by using cord blood cotinine level [6] and cotinine level in saliva during pregnancy [7]. We showed a consistent result using urinary cotinine as a biomarker of SHS exposure. A recent study has shown that maternal exposure to SHS during pregnancy measured through urine cotinine is associated with a decrease in gross motor function among children 18 months old [8]. However, no association was found for the impact of SHS exposure on cognitive function [8].

The biological mechanism by which SHS influences neurodevelopment delay has not been established yet. Huizink et al. [36] have provided potential mechanisms to explain the association between prenatal exposure to maternal smoking and neurobehavioral and cognitive outcomes based on animal and human studies. Results using rodent exposure models have suggested that prenatal nicotine exposure during critical periods of development can disrupt corticothalamic circuitry, resulting in long-lasting dysregulation of sensory information procession in the cortex [37]. A recent review study has concluded that the effect of SHS is consistent with the effect of direct smoking during pregnancy [3]. During the embryonic and fetal period, the development of central nervous system is vulnerable to toxic chemicals. Exposure to such chemicals might not only have short-term deficit, but also have long-term implications. Cigarette smoke contains thousands of noxious compounds, including mutagenic, neurotoxic, and fetotoxic agents such as nicotine, PAHs, aromatic amines, and carbon monoxide. They could pass through the placenta into the fetus [38–40]. These agents could cause fetal hypoxia-ischemia by reducing utero-placental blood flow. They might also influence fetal brain development [9]. Although cognition and motor function are known to be related to each other as the prefrontal cortex and the cerebellum may play an important role in both [41], our study demonstrated that infant’s MDI score rather than PDI score at 24 months of ages was affected by maternal SHS exposure.

Phase I and phase II metabolic enzymes are important in biotransformation of toxicants. GSTs consist of a superfamily of dimeric phase II metabolic enzymes that catalyze the conjugation of reduced glutathione with various electrophilic compounds [42]. Since GST enzymes play a vital role in cellular defense against environmentally toxic compounds, polymorphisms of GST gene can increase susceptibility to diseases caused by chemicals such as those in cigarettes. Therefore, the effect of prenatal SHS exposure on pregnancy outcomes can differ by maternal metabolic gene polymorphisms. Several studies have reported significant gene modification effects of maternal smoking or SHS exposure on birth outcomes [14–16, 43, 44]. Hsieh et al. [6] have reported that metabolic genes, GSTT1, as well as cytochrome P450 1A1 (CYP1A1) Ile462Val can modify the effect of cord blood cotinine level on early child neurodevelopment, especially for language and fine motor development using a small number of study subjects. Our present study in Korea with more participants using maternal urinary cotinine level in early pregnancy also provided evidence that metabolic genetic polymorphisms had modifying effect on the association between maternal SHS exposure in early pregnancy and neurodevelopment scores at 24 months. The proportion of double deletion of GSTM1 and GSTT1 observed in the Korean population has been found to be higher than that observed in Caucasian population, South Indians, or Afro-Americans [42]. Therefore, our study suggests the importance of considering maternal genetic polymorphisms when counseling about mother and their offspring’s health.

Breastfeeding up to 6 months is recommended by WHO because it has several health benefits [45]. It can protect against many diseases and medical conditions. It is also beneficial for the child’s development and behavior [46]. A specific examination of the relationship between breastfeeding and childhood cognition has revealed that children who are exclusively breastfed for the first 6 months have higher scores in Peabody Picture Vocabulary Test and Kaufman Brief Intelligence Test at 3 and 7 years of age than those not exclusively breastfed [47]. Some studies have suggested that the effect of breastfeeding on child neurodevelopment has to be investigated after adjusting for important confounders [48]. After controlling for some primary confounders such as maternal education level and primary caregiver during the first 24 months after birth, our study showed that the negative impact of SHS exposure at early pregnancy on MDI score at 24 months of age was diluted when children received breast milk exclusively for 6 months after birth.

We restricted mothers whose urine cotinine levels were ≤ 42.7 ng/ml and regarded them as non-active smokers because we focused on the effect of SHS exposure during pregnancy. There has been no standardized cut-off level of urinary cotinine to distinguish active smokers from non-active smokers. Several studies have suggested cut-off levels ranging from 50 to 550 ng/ml [49, 50]. These cut-off levels vary by race [51]. When we repeated analyses in all mothers without excluding mothers with high urinary cotinine levels, results were consistent (data not shown).

Previous studies have defined prenatal SHS exposed infants as those with cord blood cotinine level ≥ 0.16 ng/ml [6] or with maternal cotinine level in saliva during pregnancy ≥1.5 ng/ml [7]. Negative association was found between SHS exposure and early neurodevelopment of infants up to 24 months of age in the exposed group [6]. A negative association was also found in our study among women whose urinary cotinine levels exceeded the median of 1.90 ng/ml. However, further research is necessary concerning the threshold cotinine level in urine, saliva, or cord blood that affects neurodevelopment of infants.

The main strength of this study was its prospective cohort design with data collected from early pregnancy. We were able to study prenatal exposure in relation to children’s neurodevelopment. This prospective design enabled us to control for various potential epidemiological biases. Second, SHS exposure was not based on self-reported surveys. Instead, it was assessed using urinary cotinine as a biomarker. The use of self-reported SHS exposure might result in biased estimates because of recall bias. Several studies have described the advantage of using biomarkers for the assessment of SHS exposure compared to the use of self-reporting [52–54].

This study also has several limitations. Exposure to SHS reflects urinary cotinine level for a short time. The level of urine cotinine is associated with the amount of exposure to SHS. However, we used a single measurement of urinary cotinine level for the whole pregnancy which might not provide an accurate estimate of the exposure. Serial measurements of urinary cotinine throughout pregnancy are needed in future studies. Second, we did not include possible confounding factors such as quality of the home environment and ventilation in our analyses. Instead, we adjusted for maternal education as a surrogate marker. Third, numerous chemical compounds in SHS were activated and detoxified by both phase I and phase II enzymes. However, we could not investigate the role of other genes except GSTs due to the lack of such information in our database. Fourth, although we considered certain substantial confounders in our analyses, the effect of other environmental neurotoxins was not adjusted in our analysis. Further study is needed to investigate co-exposure of SHS and other environmental factors. Fifth, human neurodevelopment continues after birth. However, we did not consider the whole effect of postnatal exposure that could affect children’s neurodevelopment after birth except for breastfeeding behavior up to 6 months and primary caregiver up to 2 years of age. We showed that prenatal exposure to SHS with high urinary cotinine level at early pregnancy was negatively associated with neurodevelopment delay at 24 months, proposing that early pregnancy might be a critical window of public health intervention to reduce the effect. Fetal development is highly likely to be susceptible to environmental factors during the prenatal period. Thus, further analysis is needed to understand the overall association between exposure to SHS (including postnatal exposure) and children’s neurodevelopment.

## Conclusion

Exposure to maternal SHS during early pregnancy, especially in those who have high cotinine levels, may affect neurodevelopment of infants at 24 months of age. Genetic polymorphism and breastfeeding may modify the effect of SHS exposure on neurodevelopment.

## Additional file

Additional file 1: **Table S1.** Comparison of general characteristics between children with K-BSID-II scores and children without K-BSID-II scores at 24 months. **Table S2.** Association between maternal urine cotinine in early pregnancy and children’s neurodevelopment at 24 months without adjusting for creatinine level. **Table S3.** Association between maternal urine cotinine in early pregnancy and children’s neurodevelopment at 24 months stratified by genetic polymorphism and breastfeeding behavior without adjusting for creatinine level. **Figure S1.** Unadjusted associations of ln-transformed maternal urinary cotinine levels and Bayley scores in infants at 24 months of age stratified by genetic polymorphism and breastfeeding behavior. (DOCX 562 kb)

## Abbreviations

CI: Confidence Interval
GAM: Generalized Additive Model
GSTM1: Glutathione S-transferases mu 1
GSTT1: Glutathione S-transferases theta 1
K-BSID-II: Korean version of the Bayley Scales of Infant Development II
LOD: Limit of Detection
MDI: Mental Developmental Index
MOCEH: Mothers and Children Environmental Health
PAHs: Polycyclic Aromatic Hydrocarbons
PDI: Psychomotor Developmental Index
ROC: Receiver Operating Characteristic
SHS: Secondhand Smoke
WHO: World Health Organization
