# Supplementary material for: Exposure to prenatal secondhand smoke and early neurodevelopment: Mothers and Children’s Environmental Health (MOCEH) study
Source: Environ Health. 2019 Mar 20;18:22. doi: 10.1186/s12940-019-0463-9 (PMC6425627; doi:10.1186/s12940-019-0463-9)
Supplement: Supplementary file 1 — Table S1. Comparison of general characteristics between children with K-BSID-II scores and children without K-BSID-II scores at 24 months. Table S2. Association between maternal urine cotinine in early pregnancy and children’s neurodevelopment at 24 months without adjusting for creatinine level. Table S3. Association between maternal urine cotinine in early pregnancy and children’s neurodevelopment at 24 months stratified by genetic polymorphism and breastfeeding behavior without adjusting for creatinine level. Figure S1. Unadjusted associations of ln-transformed maternal urinary cotinine levels and Bayley scores in infants at 24 months of age stratified by genetic polymorphism and breastfeeding behavior. (DOCX 562 kb) [file 12940_2019_463_MOESM1_ESM.docx]

**Supplementary Tables.**

**Supplementary Table 1**. Comparison of general characteristics between children with K-BSID-II scores and children without K-BSID-II scores at 24 months.

| Characteristics | Number (%) or mean ± SD | |  |
| --- | --- | --- | --- |
|  | Children with  K-BSID-II scores  at 24 months | Children without  K-BSID-II scores  at 24 months | *P* |
| Total | 801 | 630 |  |
| **Maternal characteristics** |  |  |  |
| Maternal age, years |  |  |  |
| ≤ 30 | 475 (59.3) | 367 (58.2) | 0.69 |
| > 30 | 326 (40.7) | 263 (41.8) |  |
| Maternal education |  |  |  |
| ≤ High school | 321 (39.5) | 225 (35.7) | 1 |
| ≥ University | 408 (51.5) | 284 (45.1) |  |
| Region |  |  |  |
| Seoul | 205 (25.6) | 250 (39.7) | < 0.001 |
| Cheonan | 375 (46.8) | 153 (24.3) |  |
| Ulsan | 221 (27.6) | 227 (36.0) |  |
| GSTM1 |  |  |  |
| Present (=positive) | 386 (48.9) | 292 (47.1) | 0.51 |
| Null (=negative) | 404 (51.1) | 328 (52.9) |  |
| GSTT1 |  |  |  |
| Present (=positive) | 355 (44.9) | 262 (42.3) | 0.31 |
| Null (=negative) | 435 (55.1) | 357 (57.7) |  |
| **Infant characteristics** | |  |  |
| Infant sex |  |  |  |
| Male | 427 (53.3) | 320 (50.8) | 0.34 |
| Female | 374 (46.7) | 310 (49.2) |  |
| Breastfeeding |  |  |  |
| Yes | 283 (35.3) | 114 (18.1) | 0.23 |
| No | 431 (53.8) | 207 (32.9) |  |
| Primary caregiver during the first 24 months after birth | |  |  |
| Mother | 408 (50.9) | 35 (5.6) | < 0.001 |
| Others | 393 (49.1) | 595 (94.4) |  |
| Gestational Age | 38.84 ± 1.53 | 38.78 ± 1.77 | 0.45 |

**[Incl. Mothers whose urinary cotinine level at early pregnancy was less than 42.7 ng/ml but without adjusting for creatinine level]**

**Supplementary Table 2.** Association between maternal urine cotinine in early pregnancy and children’s neurodevelopment at 24 months

| Cotinine | Bayley | n | Mean±SD | Unadjusted^1^ | | | Adjusted ^2^ | | |
| --- | --- | --- | --- | --- | --- | --- | --- | --- | --- |
|  |  |  |  | β | 95% C.I. | *P* | β | 95% C.I. | *P* |
| ≤ **1.90** | MDI | 368 | 97.05±14.81 | 1.41 | (-1.57 , 4.38) | 0.35 | 1.17 | (-1.66 , 4.01) | 0.42 |
|  | PDI | 368 | 96.86±14.07 | 1.63 | (-1.20 , 4.46) | 0.26 | 1.70 | (-1.06 , 4.47) | 0.23 |
| > **1.90** | MDI | 352 | 96.97±14.24 | -1.79 | (-4.29 , -0.71) | 0.16 | -2.30 | (-4.72 , 0.11) | 0.06 |
|  | PDI | 352 | 96.70±12.89 | -0.60 | (-2.87 , 1.67) | 0.61 | -0.63 | (-2.82 , 1.57) | 0.58 |

1 General Linear Model.

2 General Linear Model adjusted for maternal age, maternal education, gestational age, infant sex, region, breastfeeding behavior, and primary caregiver.

MDI: Mental Development Index, PDI: Psychomotor Development Index

**Supplementary Table 3.** Association between maternal urine cotinine in early pregnancy and children’s neurodevelopment at 24 months stratified by genetic polymorphism and breastfeeding behavior

|  | **Cotinine** | **≤ 1.90^1^** | | | | **> 1.90^1^** | | | |
| --- | --- | --- | --- | --- | --- | --- | --- | --- | --- |
|  |  | **n** | **β^2^** | **95% C.I.** | ***P*** | **n** | **β^2^** | **95% C.I.** | ***P*** |
| MDI | GSTM1/GSTT1 |  |  |  |  |  |  |  |  |
|  | Any present | 241 | 2.43 | (-1.20 , 6.06) | 0.19 | 251 | -1.26 | (-4.24 , 1.72) | 0.40 |
|  | Both null | 119 | -0.64 | (-5.78 , 4.49) | 0.80 | 98 | **-4.99** | **(-9.47, -0.51)** | **0.03** |
|  | Breastfeeding only up to 6 months^3^ | |  |  |  |  |  |  |  |
|  | Yes | 135 | 0.0005 | (-4.72 , 4.72) | > 0.999 | 122 | 0.24 | (-3.90 , 4.39) | 0.91 |
|  | No | 195 | 2.29 | (-1.94 , 6.51) | 0.29 | 188 | -3.26 | (-6.68 , 0.17) | 0.06 |
| PDI | GSTM1/GSTT1 |  |  |  |  |  |  |  |  |
|  | Any present | 241 | 2.19 | (-1.30 , 5.68) | 0.22 | 251 | -0.92 | (-3.70 , 1.85) | 0.51 |
|  | Both null | 119 | 2.16 | (-3.01 , 7.32) | 0.41 | 98 | -1.16 | (-5.14, 2.83) | 0.57 |
|  | Breastfeeding only up to 6 months^3^ | |  |  |  |  |  |  |  |
|  | Yes | 135 | -0.75 | (-5.85 , 4.35) | 0.77 | 122 | 1.11 | (-3.00 , 5.22) | 0.59 |
|  | No | 195 | 3.19 | (-0.60 , 6.98) | 0.10 | 188 | -1.37 | (-4.38 , 1.63) | 0.37 |

1 median of urinary cotinine level = 1.90 (ng/ml)

2 General Linear Model adjusted for maternal age, maternal education, gestational age, infant sex, region, breastfeeding behavior, and primary caregiver.

3 General Linear Model adjusted for maternal age, maternal education, gestational age, infant sex, region, and primary caregiver.

MDI: Mental Development Index, PDI: Psychomotor Development Index, GSTM1 : Glutathione S-transferases mu1, GSTT1 : Glutathione S-transferases theta 1

**Supplementary Figure**

**GSTM1/GSTT1 Breastfeeding up to 6 months**

Unadjusted associations of ln-transformed maternal urinary cotinine levels and Bayley scores in infants at 24 months of age stratified by genetic polymorphism and breastfeeding behavior. The Generalized Additive Model was used**.** Dashed vertical straight line at 0.64 in each figure represents the median level of urinary cotinine, exp(0.64) =1.90.

MDI: Mental Development Index; PDI: Psychomotor Development Index; GSTM1: Glutathione S-transferases mu1; GSTT1: Glutathione S-transferases theta 1.
